# Higher aggression towards closer relatives by soldier larvae in a polyembryonic wasp

**DOI:** 10.1098/rsbl.2014.0229

**Published:** 2014-05

**Authors:** Johanna Dunn, Derek W. Dunn, Michael R. Strand, Ian C. W. Hardy

**Affiliations:** 1 School of Biosciences, University of Nottingham, Sutton Bonington, UK; 2 College of Life Sciences, Northwest University, Xi'an, Shaanxi 710069, People's Republic of China; 3 Department of Entomology, University of Georgia, Athens, GA, USA

**Keywords:** aggression, relatedness, polyembryonic wasps

## Abstract

In the polyembryonic wasp *Copidosoma floridanum*, females commonly lay one male and one female egg in a lepidopteran host. Both sexes proliferate clonally within the growing host larva. Distinct larval castes develop from each wasp egg, the majority being ‘reproductives’ plus some ‘soldiers’ which sacrifice reproduction and attack competitors. Maturing mixed sex broods are usually female biased, as expected when intra-brood mating is common. Pre-mating dispersal followed by outbreeding is expected to increase sexual conflict over brood sex ratios and result in greater soldier attack rates. Owing to sexually asymmetric relatedness, intra-brood conflicts are expected to be resolved primarily via female soldier attack. We observed soldier behaviour *in vitro* to test whether lower intra-brood relatedness (siblings from either within-strain or between-strain crosses were presented) increased inter-sexual aggression by female as well as male soldiers. As found in prior studies, females were more aggressive than males but, contrary to expectations and previous empirical observations, soldiers of both sexes showed more aggression towards more closely related embryos. We speculate that lower intra-brood relatedness indicates maternal outbreeding and may suggest a rarity of mating opportunities for reproductives maturing from the current brood, which may enhance the value of opposite sex brood-mates, or that higher aggression towards relatives may be a side-effect of mechanisms to discriminate heterospecific competitors.

## 1. Introduction

The degree of relatedness between individuals underpins much of social evolution theory [1]. For instance, indirect genetic benefits gained by high relatedness can predict increased likelihood of altruism, even when cooperation involves direct costs. Conversely, aggression will more likely occur between more distantly (or negatively) related individuals, even when this behaviour incurs costs to the actor, i.e. spite [2]. This is most likely to occur in outbred sub-populations in which relatedness between interacting individuals is low. With reduced dispersal between sub-populations and hence increased rates of inbreeding, relatedness increases within sub-populations, meaning that aggression rates are predicted to be reduced [2]. However, these predictions are compounded because increased relatedness in sub-populations also often increases the level of costs of aggression/altruism associated with competition for local resources [3,4]. This leads to difficulty in differentiating between the effects of these two important predictors of social behaviour.

The unusual biology of some encyrtid polyembryonic parasitoid wasps enables the effects of relatedness on aggression to be manipulated experimentally, while costs associated with competition for host resources remain relatively unchanged, i.e. host size [4]. In *Copidosoma floridanum*, females commonly lay one male and one female egg in a lepidopteran host egg. Both sexes proliferate clonally within the growing host larva. Two distinct larval castes develop from each wasp egg, the majority being ‘reproductives’ plus some ‘soldiers’ which sacrifice reproduction and attack heterospecific and conspecific competitors [5–8]. Maturing mixed sex broods are usually female biased, as expected when intra-brood mating is common (local mate competition [9]). Pre-mating dispersal followed by outbreeding is expected to increase sexual conflict over brood sex ratios [2] and, in general, increased conflicts can result in greater numbers of soldiers being produced [6], and higher probabilities of attack by individual soldiers [4,10]. Owing to sexually asymmetric relatedness, intra-brood conflicts are expected to be resolved primarily via female soldier attack [2].

We observed soldier behaviour *in vitro* to test whether lower intra-brood relatedness (manipulated by whether or not the father was from the maternal sub-population) increased inter-sexual aggression by female as well as male soldiers. To do this, we used two experiments each using as potential conspecific competitors either: (i) polymorula (tissue that becomes ‘reproductive’ larvae) or (ii) soldiers.

## 2. Material and methods

We used two *C. floridanum* sub-populations that both originated from the USA. (i) A ‘lab’ strain that had been maintained in the laboratory for over 20 years since original field collection in Sumter County, SC, USA [5]. (ii) A ‘field’ strain, collected specifically for this experiment from Tift County, GA, USA in autumn 2011. This was maintained in the laboratory immediately prior to and during the experiment.

### (a) Experiment 1

**Figure 1.**
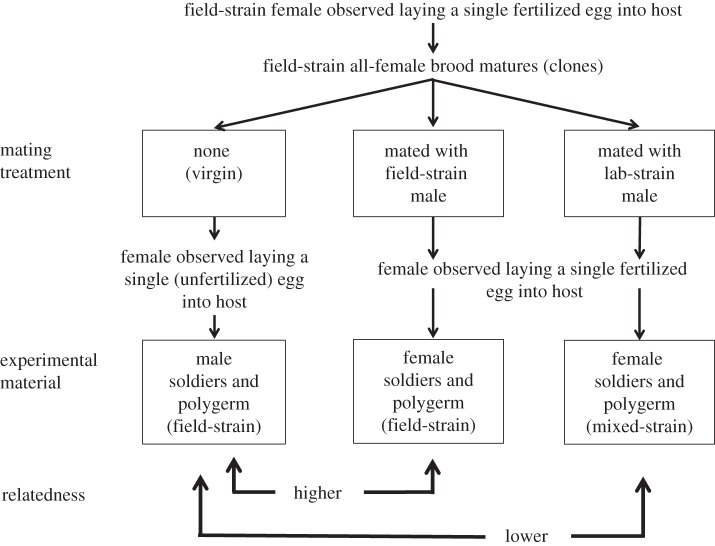
Schematic summary of the method used to prepare biological material of varying relatedness.

In the Hymenoptera, males are haploid with all genes deriving from their mother, whereas females are biparentally diploid [11]. We let virgin ‘field’ females from a given brood lay male eggs, meanwhile mating their (genetically identical) sisters from the same brood with either ‘field’ males or ‘lab’ males. We thus created broods with higher relatedness (within-strain cross) or lower relatedness (between-strain cross) to broods of males produced by the virgin mothers (figure 1). As the virgin and mated mothers were identical twin sisters, owing to polyembryonic development, these ‘cousin’ broods were genetically equivalent to ‘sibling’ broods. Identifying the sexual composition of broods produced by mated mothers is possible because females exhibit different behaviours when laying a fertilized or an unfertilized egg [5]. When host larvae had reached their fourth instar they were dissected, and soldiers and polymorulae (developing ‘reproductives’) were removed for *in vitro* experimentation.

In each replicate, a female or a male soldier of known brood and strain origin was placed in a culture-well containing 50 µl of TC-100 medium with an opposite sex polymorula, also of known origin (table 1). Hence, soldier sex and soldier–polymorulae relatedness were varied in a two-way factorial design. Each soldier was observed with the aid of a stereomicroscope at 20× magnification. Soldier aggression was scored as occurring if the soldier attacked the polymorula, by biting, within a 1 h observation period. Aggressive attack behaviour was distinguished by a soldier's head lurching towards the polygerm while clearly biting with its mandibles.

**Table 1.** Sample sizes of soldier–polymorula encounters.

origin of female brood	soldier sex in trial		
	male	female	total
within-strain cross	58	64	122
between-strain cross	57	65	122
total	115	129	

### (b) Experiment 2

We also performed an experiment to test whether soldiers attack opposite sex soldiers originating from same strain (field versus field; *n* = 45) or from a different strain (field versus laboratory; *n* = 18). In each replicate, a focal soldier was placed in a culture-well containing 50 µl of TC-100 medium with an opposite sex (target) soldier, and then observed for 1 h. We stained the target soldier in each pair with carboxy-flourescein diacetate succinimidyl ester dye in order to differentiate between morphologically identical soldiers. Focal soldier aggression was assessed as described above, and by the detection of labelled material ingested during an attack by the use of an epiflourescent microscope [4].

### (c) Statistical analyses

We calculated the probability of soldier attack using logistic analyses (GenStat, v. 15.1, VSN International Ltd.), with backwards elimination of non-significant explanatory variables [12]. We first used logistic factorial ANOVA (a generalized linear model, GLM), treating each of the 244 replicates as an independent binary observation (1 = soldier attacked, 0 = soldier did not attack) and assumed binomially distributed errors, the results of which are reported below.

Owing to practical constraints on biological material availability, not every soldier or polymorula used in the experiment was independent in origin to every other. For instance, sometimes several soldiers were taken from the same brood for use in separate replicates. We accounted for the potential for this pseudo-replication to generate false significance (type I error) in two ways. First, we grouped replicates into 48 unique combinations of soldier origin plus polymorula origin, and then performed logistic analysis on the number of soldiers that attacked per group. The interpretation of these results was identical to those from analysis of the ungrouped data (see the electronic supplementary material). Because the groupings did not entirely eliminate members of the same brood appearing in multiple rows of the dataset, we further carried out generalized linear mixed modelling [12] with the origins of the biological material fitted as random effects, and soldier sex and relatedness fitted as fixed effects: this also confirmed the interpretation of the initial analysis (see the electronic supplementary material). Here we report results from the initial GLM.

## 3. Results

**Figure 2.**
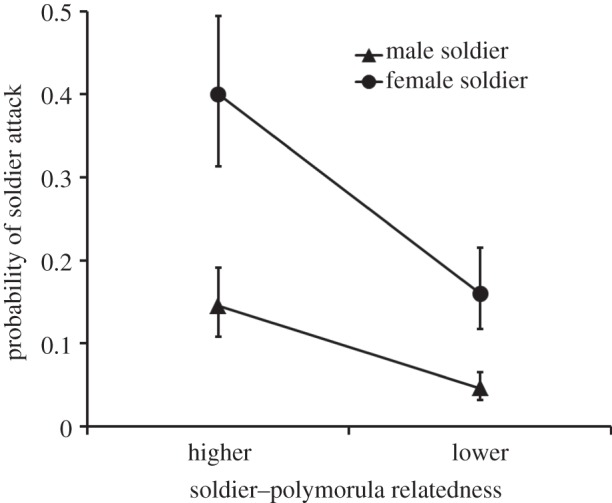
Attack rate of soldiers against opposite sex polymorulae (±s.e). The convergence of the lines is due to back-transformation of estimated proportions from the logit scale (on which they are parallel) and not due to a statistical interaction (which was non-significant, see main text).

Female soldiers were more likely to attack polymorulae than were male soldiers (*G*_1_ = 14.74, *p* < 0.001; figure 2). Soldiers of both sexes showed more aggression towards more closely related polymorulae than towards less closely related polymorulae (*G*_1_ = 12.88, *p* < 0.001; figure 2.). There was no significant interaction effect between soldier sex and soldier relatedness to the polymorula (*G*_1_ = 2.16, *p* = 0.14). In our second experiment involving only soldiers, none attacked any other soldier regardless of sex or relatedness (statistical analyses are thus not presented).

## 4. Discussion

Our data concur with previous reports showing female *C. floridanum* soldiers are more aggressive than males [4], and are more likely to attack the polymorula of an intra-specific competitor than are males [10]. This is consistent with one function of *C. floridanum* soldiers being to mediate sex ratio conflict within mixed sex broods in favour of females [2]. This is due to the genetic asymmetry of haplodiploid sex determination between brothers, sisters and their mother: under outbreeding the genetic interests of full-sibling sisters align most closely and mother–son relatedness is sexually asymmetric [1] but inbreeding increases mother–daughter, but not mother–son, relatedness, making females more valuable and hence a reduction in males within broods is predicted [2,13]; inbreeding is also associated with local mate competition which also selects for female bias within broods [9]. It has been suggested that male soldiers exist only as a non-adaptive side-effect of the evolution of female soldiers [7,10]. Our finding that male soldiers have the same relatively low aggression rates towards female competitors thus supports in part a non-adaptive male soldier function.

That male and female soldiers were more likely to attack closer relatives is contrary to expectations and previous empirical observations [4]. While male soldier aggression is much lower than that of females, these data also add to recent evidence showing that male soldiers are not entirely passive [14]. Intra-specific competition may be higher between close relatives than more distantly related individuals, which may explain why aggression was shown towards close relatives, but a mechanism for this is unclear.

Female *C. floridanum* soldiers occur as two types that differ in both morphology and behaviour. Those that emerge prior to hatching of a host egg are extremely small and immobile, which results in these individuals primarily attacking conspecifics [7]. By contrast, soldiers that emerge during the host larval stage are much larger and more mobile, which are traits needed for attacking heterospecific competitors [6,7]. Short, early emerging female soldiers may thus be more able to adaptively detect variation between two potential conspecific competitors and *vice versa*. As our experimental procedure was constrained to use longer, later emerging soldiers, higher aggression towards relatives may thus have been a non-adaptive side-effect of mechanisms that enable discrimination between different species of heterospecific competitors: this requires further research as other species of polyembryonic wasps in which sex ratio conflict rarely occurs produce soldiers of both sexes that aggressively attack heterospecific competitors but weakly attack conspecifics [15].

In the field, wasp broods comprising all female offspring derived from multiple foundresses are extremely rare, consistent with the soldiers of a female ‘resident’ eliminating competitors prior to hatching [7]. When all-male broods occur in the field, the probability of the offspring being derived from multiple foundresses is higher than for all female broods [7], consistent with fewer (and less aggressive) soldiers being present. This suggests that interactions between relatively distantly related soldiers in the field may be very rare, and the patterns we observed may have been due to other, unknown mechanisms. Alternatively, because there is variation in host density and availability, mating opportunities for single sex *C. floridanum* broods can be scarce [5,16]. We speculate that lower intra-brood relatedness indicates maternal outbreeding and may suggest a rarity of mating opportunities for reproductives maturing from the current brood, which may thus enhance the value of opposite sex brood-mates. Further understanding of solider behaviour in polyembryonic parasitoids will require additional investigation into their roles in inter- and intra-specific competition and also into the mechanisms underlying how soldiers recognize potential competitors [4,6,7,8,10].

## Data accessibility

The datasets supporting this article have been uploaded as part of the electronic supplementary material.
